# Supplementary material for: Cysteine-rich intestinal protein 1 is a novel surface marker for human myometrial stem/progenitor cells
Source: Commun Biol. 2023 Jul 3;6:686. doi: 10.1038/s42003-023-05061-0 (PMC10317972; doi:10.1038/s42003-023-05061-0)
Supplement: Supplementary file 2 — Supplementary Information [file 42003_2023_5061_MOESM2_ESM.pdf]

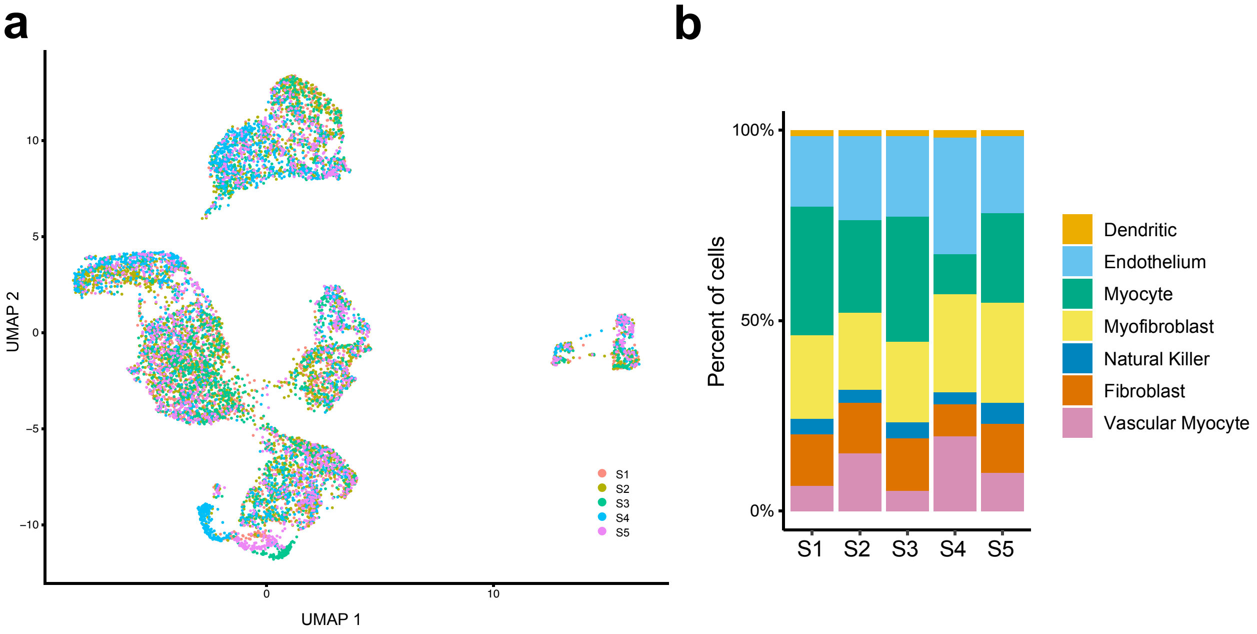

Supplementary Figure 1. Cell distribution across cell clusters in the single cell RNA-seq.

(a) Uniform manifold approximation and projection (UMAP) visualization of 9775 isolated cells from human myometrial samples (n = 5). Each color dot represents cells from a myometrials from a different patient. UMAP plot shows that each patient's cells are well distributed across clusters. **(b)** Cell proportion of each cluster as a percentage across patients.

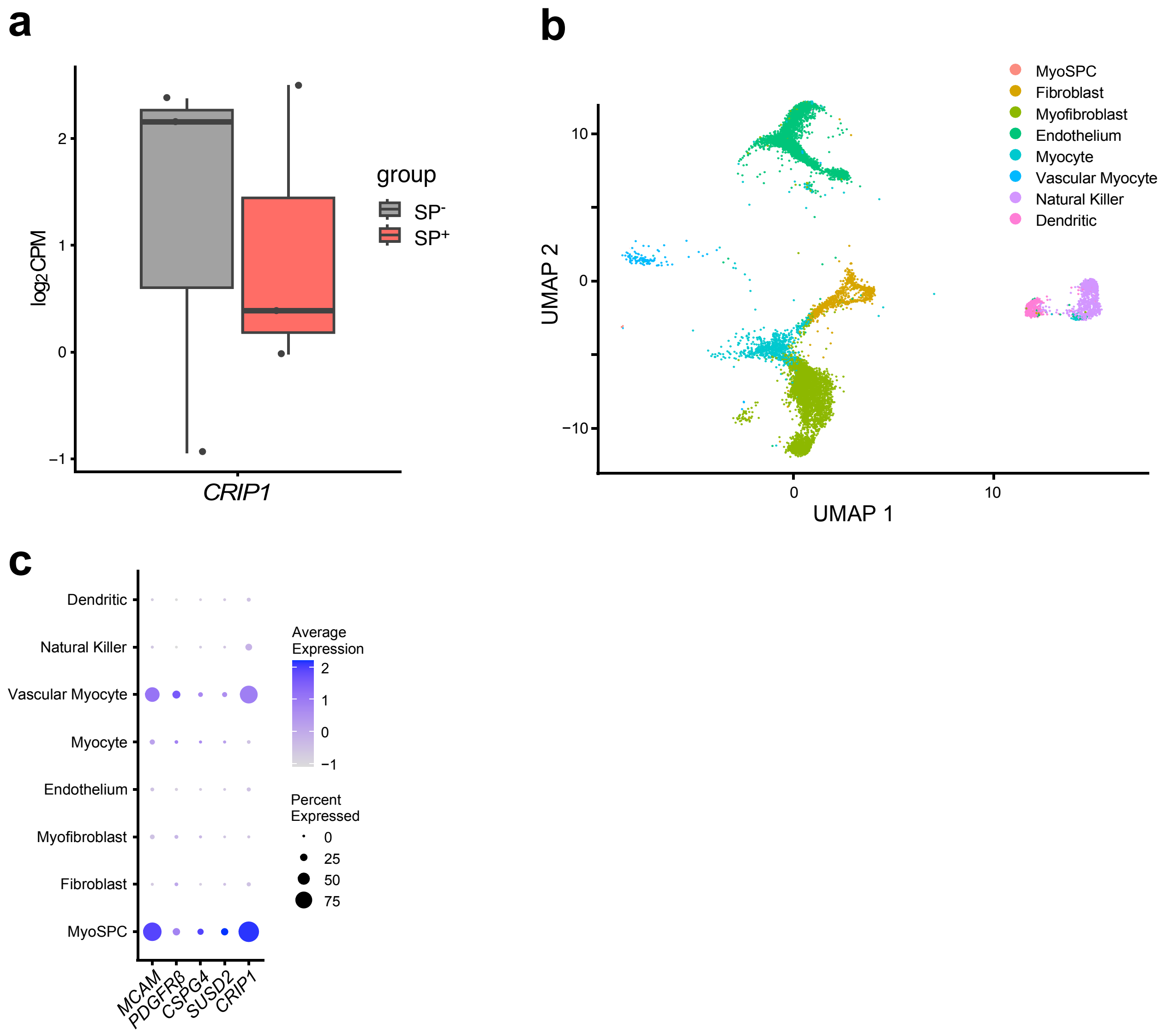

Supplementary Figure 2. CRIP1 expression in the side population and an orthogonal single cell study.

(a) CRIP1 expression in log_2CPM of the RNA-seq results from the SP+ is not significantly different from that of the SP- cells (FDR>0.05). **(b)** Projection of a data set of 18,939 cells from 5 myometrial samples from fibroids patients (38) onto the UMAP in Fig 3A. **(c)** Dotplot of mesenchymal stem cell markers and CRIP1 gene expression in the different myometrial cell clusters as defined in Fig 3C.

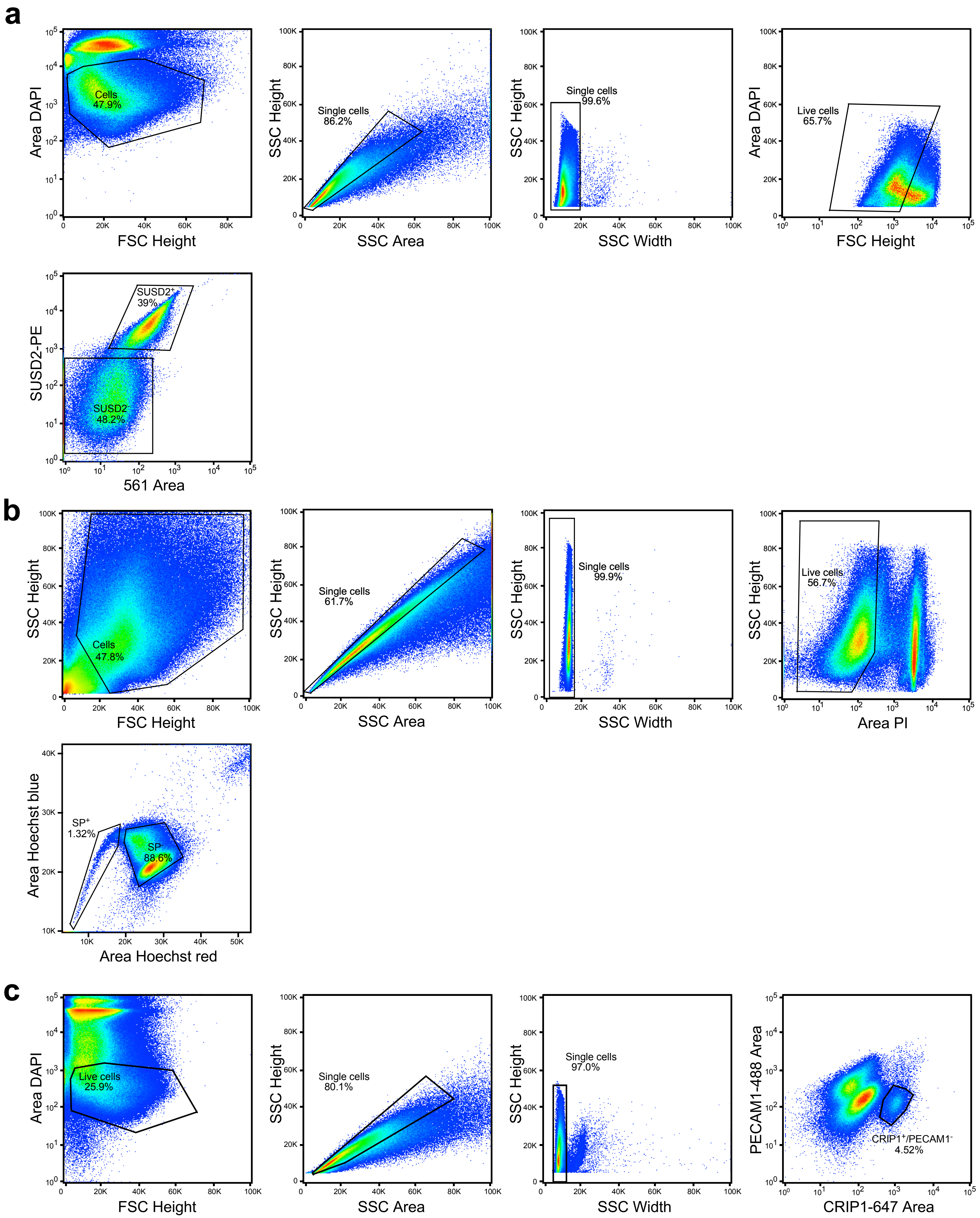

Supplementary Figure 3. Gating strategy used for cell sort.
Example of the gating strategy of **(a)** SUSD2+ and SUSD2-, **(b)** side population (SP) SP+ and SP-, and **(c)** CRIP1+/PECAM1- cell sort.
