# Supplementary material for: Cysteine-rich intestinal protein 1 is a novel surface marker for human myometrial stem/progenitor cells
Source: Commun Biol. 2023 Jul 3;6:686. doi: 10.1038/s42003-023-05061-0 (PMC10317972; doi:10.1038/s42003-023-05061-0)
Supplement: Supplementary file 3 — Description of Additional Supplementary Files [file 42003_2023_5061_MOESM3_ESM.pdf]

Description of Additional Supplementary Files

File name: Supplementary Data 1
Description: Differentially expressed genes between SUSD2+ and SUSD2- myometrial cells with a false discovery rate (FDR) adjusted p-value <0.05

File name: Supplementary Data 2
Description: Differentially expressed genes between SP+ and SP- myometrial cells with a false discovery rate (FDR) adjusted p-value <0.05

File name: Supplementary Data 3
Description: Differentially expressed genes between MyoSPC cluster and the remaining myometrial cells with a false discovery rate (FDR) adjusted p-value <0.05

File name: Supplementary Data 4
Description: Overlapping differentially expressed genes in MyoSPC remaining myometrial cells and SUSD2+/- cells comparisons with a false discovery rate (FDR) adjusted p-value <0.05

File name: Supplementary Data 5
Description: Cell distributions in clusters in current study and in Goad et al,[38]

File name: Supplementary Data 6
Description: Percentage of colonies calculated by the number of colonies formed divided by the number of cells seeded multiplied by 100 in CRIP1+/PECAM- and depleted myometrial cells (Figure 6d)

File name: Supplementary Data 7
Description: Total area of colony formed in pixels from CRIP1+/PECAM- and depleted myometrial cells (Figure 6e)

File name: Supplementary Video
Description: 3D imaging of a myometrium sample showing that CRIP1+ cells (green) are surrounding the PECAM1+ (red) vascular endothelial cells. DAPI staining (white) was use to stain nuclei. Scale bar is shown on the bottom left
