# Supplementary material for: Cysteine-rich intestinal protein 1 is a novel surface marker for human myometrial stem/progenitor cells
Source: Commun Biol. 2023 Jul 3;6:686. doi: 10.1038/s42003-023-05061-0 (PMC10317972; doi:10.1038/s42003-023-05061-0)
Supplement: Supplementary file 6 — Reporting Summary [file 42003_2023_5061_MOESM6_ESM.pdf]

nature portfolio

Corresponding author(s): COMMSBIO-23-1323-T

Last updated by author(s): 04-25-2023

Reporting Summary

Nature Portfolio wishes to improve the reproducibility of the work that we publish. This form provides structure for consistency and transparency in reporting. For further information on Nature Portfolio policies, see our Editorial Policies and the Editorial Policy Checklist.

Statistics

For all statistical analyses, confirm that the following items are present in the figure legend, table legend, main text, or Methods section.

n/a	Confirmed	
☐	☒	The exact sample size (*n*) for each experimental group/condition, given as a discrete number and unit of measurement
☐	☒	A statement on whether measurements were taken from distinct samples or whether the same sample was measured repeatedly
☐	☒	The statistical test(s) used AND whether they are one- or two-sided<br>*Only common tests should be described solely by name; describe more complex techniques in the Methods section.*
☒	☐	A description of all covariates tested
☐	☒	A description of any assumptions or corrections, such as tests of normality and adjustment for multiple comparisons
☐	☒	A full description of the statistical parameters including central tendency (e.g. means) or other basic estimates (e.g. regression coefficient) AND variation (e.g. standard deviation) or associated estimates of uncertainty (e.g. confidence intervals)
☒	☐	For null hypothesis testing, the test statistic (e.g. *F*, *t*, *r*) with confidence intervals, effect sizes, degrees of freedom and *P* value noted<br>*Give P values as exact values whenever suitable.*
☒	☐	For Bayesian analysis, information on the choice of priors and Markov chain Monte Carlo settings
☒	☐	For hierarchical and complex designs, identification of the appropriate level for tests and full reporting of outcomes
☒	☐	Estimates of effect sizes (e.g. Cohen's *d*, Pearson's *r*), indicating how they were calculated

Our web collection on statistics for biologists contains articles on many of the points above.

Software and code

Policy information about availability of computer code

Data collection	Bulk RNA-seq: Libraries were prepared using a Kapa RNA HyperPrep kit with ribosomal reduction, pooled, and sequenced on on an Illumina NextSeq 6000 instrument.<br><br>Single cell RNA-seq: Libraries were generated and sequenced using the 10X Chromium Next GEM Single Cell 3′ GEM kit (10X Genomics, v2) platform. Paired end sequencing was performed on an Illumina NovaSeq 6000 sequencer using an S2 flow cell, 100 cycle sequencing kit (v1.5) to a minimum depth of 50K reads per cell (Illumina Inc., San Diego, CA, USA). Base calling was done by Illumina RTA3 and output was demultiplexed and converted to FastQ format with Cell Ranger (10X Genomics, v3.1.0).
Data analysis	Bulk RNA-seq: Reads were trimmed using TrimGalore (version 0.6.5), and mapped to Homo sapiens genome assembly GRCh38 (hg38) using STAR (version 2.7.9a). Reads overlapping Ensembl annotations (version 99) were quantified with STAR prior to model-based differential expression analysis using the edgeR-robust method. Scatterplots of two selected principal components was constructed with the PCAtools R package (version 2.5.13) to verify sample separation prior to statistical testing. Genes were considered differentially expressed if their respective edgeR-robust FDR corrected p-values were less than 0.05.<br><br>Single cell RNA-seq: Reads were aligned to the Homo sapiens genome assembly GRCh38 (hg38) using STAR (version 2.7.9a) with 10X Genomics Cell Ranger (version 3.1.0). Samples were merged using the integration anchors function of the Seurat package (version 4.2.1) from R. Genes expressed in fewer than three cells in a sample were excluded, as well as cells that expressed fewer than 200 genes and mitochondrial gene content >5% of the total unique molecular identifier count. Data were normalized using a global-scaling normalization method that normalizes the feature expression measurements for each cell by the total expression, multiplies this by a scale factor (10,000), and then log-transforms the results. The top 2,000 most variable genes that were used for cell clustering were found using the FindVariableFeatures function and were then normalized using the ScaleData function. Based on an elbow plot generated using the

Elbowplot function of Seurat, we selected 15 principal components for downstream analyses. Cell clusters were generated using FindNeighbors and FindClusters functions. Cell cycle score and velocity were determined using the functions CellCycleScoring from Seurat and RunVelocity from SeuratWrappers (version 0.3.0), respectively. The "stem cell" cluster was selected using the CellSelector function from Seurat.

For manuscripts utilizing custom algorithms or software that are central to the research but not yet described in published literature, software must be made available to editors and reviewers. We strongly encourage code deposition in a community repository (e.g. GitHub). See the Nature Portfolio guidelines for submitting code & software for further information.

Data

Policy information about availability of data

All manuscripts must include a data availability statement. This statement should provide the following information, where applicable:

- Accession codes, unique identifiers, or web links for publicly available datasets
- A description of any restrictions on data availability
- For clinical datasets or third party data, please ensure that the statement adheres to our policy

Raw fastq files were deposited in the NCBI Gene Expression Omnibus (GSEXXXX).

Research involving human participants, their data, or biological material

Policy information about studies with human participants or human data. See also policy information about sex, gender (identity/presentation), and sexual orientation and race, ethnicity and racism.

Reporting on sex and gender	Samples used in this study were myometrium tissues from self-identified women
Reporting on race, ethnicity, or other socially relevant groupings	Samples used in this study were myometrium tissues from self-identified Caucasian
Population characteristics	Samples were from non-fibroid pre-menopausal patients from 34 to 50 years old.
Recruitment	*Describe how participants were recruited. Outline any potential self-selection bias or other biases that may be present and how these are likely to impact results.*
Ethics oversight	Human tissue specimens was approved by the Spectrum Health Systems Institutional Review Board as secondary use of biobank materials

Note that full information on the approval of the study protocol must also be provided in the manuscript.

Field-specific reporting

Please select the one below that is the best fit for your research. If you are not sure, read the appropriate sections before making your selection.

☒ Life sciences ☐ Behavioural & social sciences ☐ Ecological, evolutionary & environmental sciences

For a reference copy of the document with all sections, see nature.com/documents/nr-reporting-summary-flat.pdf

Life sciences study design

All studies must disclose on these points even when the disclosure is negative.

Sample size	For bulk and single cell RNA-seq 5 myometrial samples were use to reduce risk of patient variability. For confirming CRIP1 as a stem cell marker, a separate set of patient n>3 was use as a minimum number of technical needed for statistical rigor.
Data exclusions	No data were exclude from the analysis
Replication	A separate set of tissue samples were use to confirm CRIP1 stem cell activity. In this study we also use single-cell RNA-seq data from a published study and confirmed that CRIP1 expression was found in the Myometrium Stem Cell cluster (GSE162122).
Randomization	Samples use for this study was collected by Spectrum Heath and our criteria for selecting samples was pre-menopausal patients without fibroids or any hormonal treatment.
Blinding	No information of the patient was given to us.

Reporting for specific materials, systems and methods

We require information from authors about some types of materials, experimental systems and methods used in many studies. Here, indicate whether each material, system or method listed is relevant to your study. If you are not sure if a list item applies to your research, read the appropriate section before selecting a response.

Materials & experimental systems

n/a	Involved in the study
☐	☒ Antibodies
☒	☐ Eukaryotic cell lines
☒	☐ Palaeontology and archaeology
☒	☐ Animals and other organisms
☒	☐ Clinical data
☒	☐ Dual use research of concern
☒	☐ Plants

Methods

n/a	Involved in the study
☒	☐ ChIP-seq
☐	☒ Flow cytometry
☒	☐ MRI-based neuroimaging

Antibodies

Antibodies used	SUSD2-PE anti-human (Miltenyi Biotec, #130-117-682, clone: W5C5, lot:5201112116), PECAM1-FITC anti-human (ThermoFisher, #11-0319-42, clone: WM59, lot:2171939), CRIP1 rabbit anti-human (ThermoFisher, #PA5-24643, lot:XA3473606), PECAM1 (Abcam, #ab9498, clone: JC/70A), Alexa Fluor 555 (Invitrogen, #A31572, lot:2482963), Alexa Fluor 647 (Invitrogen, #A21235, lot: 2277746) and αSMA-Cy3 (Sigma, #C6198, clone: 1A4, lot: 042M4779).
Validation	SUSD2-PE: PMID: 24964924<br>PECAM1-FITC: The WM-59 (WM59) antibody has been reported for use in flow cytometric analysis. The antibody has been pre-titrated and tested by flow cytometric analysis of normal human peripheral blood cells. This can be used at 5 µL (0.5 µg) per test. A test is defined as the amount (µg) of antibody that will stain a cell sample in a final volume of 100 µL. Cell number should be determined empirically but can range from 10^5 to 10^8 cells/test<br>CRIP1: Antibody tested by manufacturer by flow cytometry analysis of HL-60 cells<br>PECAM1 (abcam): tested and suitable for immuno-histochemistery, PMID: 33621431<br>αSMA-Cy3: tested for immunofluorescence in multiple peer-reviewed papers, including PMID 24885166

Flow Cytometry

Plots

Confirm that:

☒ The axis labels state the marker and fluorochrome used (e.g. CD4-FITC).

☒ The axis scales are clearly visible. Include numbers along axes only for bottom left plot of group (a 'group' is an analysis of identical markers).

☒ All plots are contour plots with outliers or pseudocolor plots.

☐ A numerical value for number of cells or percentage (with statistics) is provided.

Methodology

Sample preparation	Human primary myometrial cells were thawed and resuspended in 1% bovine serum albumin blocking buffer for 20 min at room temperature (RT). Cells were then incubated with the primary antibody for 45 min at RT; SUSD2-PE anti-human (Miltenyi Biotec, #130-117-682), PECAM1-FITC anti-human (ThermoFisher, #11-0319-42), and CRIP1 rabbit anti-human (ThermoFisher, #PA5-24643). For CRIP1/PECAM1 staining, cells were incubated with an Alexa-647 anti-rabbit secondary antibody for 30 min at RT. Stained myometrial cells were then wash with flow buffer and resuspend in 1 mL of flow buffer with 1 µg of 4′,6-diamidino-2-phenylindole (DAPI) or Propidium Iodode (PI), depending on the experiment, for live dead discrimination.<br><br>The side population assay live cells were incubated with 5 µg/mL of Hoechst 33342 dye for 90 min. As a negative control, separate aliquots of cells from the same patients were treated with 25 µg/ml of verapamil prior to addition of the Hoechst dye. PI was added to stained cells with and without verapamil treatment.<br><br>Cells were sorted by the flow cytometry core at Van Andel Research Institute (VARI).
Instrument	FACSymphony S6 cytometer (BD Biosciences) and MoFlo Astrios (Beckman Coulter)
Software	FlowJo Software (BD Biosciences, version 10.8.1)
Cell population abundance	For purity quality, sorted cells from one sample were ran into the flow cytometry machine to check that all cells were live and PE/647 positifs.
Gating strategy	Control samples were run into the FACS before running the full stained sample: DAPI or PI only, and secondary antibody (alexa) only.<br>Live cells were gated using the DAPI Area vs 488-FSC-Height scatter plot.

Single cells were gated using 488-SSC-area vs 488-SSC-Height and 488-SSC-withvs 488-SSC-Height.
CRIP1+/PECAM- cells were gated using 640-671/30-Area vs 488-510/120-area

☒ Tick this box to confirm that a figure exemplifying the gating strategy is provided in the Supplementary Information.
